# Nucleus accumbens GLP-1 signaling and its role in reward and metabolic regulation: a systematic review

**DOI:** 10.3389/fnana.2026.1880560

**Published:** 2026-07-16

**Authors:** Maysa Edwiges, Susana I. Sá, Rui Lopes, Cesar Portela, José Paulo Andrade, João Rocha-Neves

**Affiliations:** 1 Faculty of Medicine, University of Porto, Porto, Portugal; 2 RISE-Health, Faculty of Medicine, University of Porto, Porto, Portugal; 3 Department of Psiquiatry, Hospital Lusíadas – Braga, Braga, Portugal; 4 Unidade de Alcoologia do Porto, Matosinhos, Portugal; 5 Unidade de Desabituação do Norte, Matosinhos, Portugal; 6 Department of Vascular Surgery, Unidade Local de Saúde do Alto Ave (ULSAAVE), Guimarães, Portugal

**Keywords:** dopaminergic neurons, feeding behaviour, glucagon-like peptide-1 receptor, neural pathways, reward system

## Abstract

**Introduction:**

Glucagon-like peptide-1 (GLP-1) is an incretin hormone involved in glucose homeostasis, appetite regulation, and energy balance. Beyond its peripheral metabolic actions, growing evidence indicates that GLP-1 signalling within the central nervous system modulates reward-related processes. The nucleus accumbens (NAc), a key component of the mesolimbic reward pathway, represents a critical site where metabolic and motivational signals converge. However, the role of GLP-1 signalling within the human NAc remains incompletely understood. This systematic review aimed to synthesize current human evidence on GLP-1 expression and receptor signalling in the NAc and their role in reward and metabolic regulation.

**Methods:**

A systematic search of PubMed, Scopus, and Web of Science was conducted in December 2025. Human studies assessing GLP-1 or GLP-1 receptor (GLP-1R) in relation to NAc structure, function, or connectivity were included. Study selection and data extraction were performed independently by two reviewers.

**Results:**

From 276 screened records, five studies were included, totalling 284 participants. No consistent evidence emerged for direct GLP-1-related alterations in isolated NAc activation or GLP-1R expression. Instead, GLP-1 modulation of mesolimbic connectivity, particularly between the NAc and orbitofrontal cortex (OFC), was observed.

**Discussion:**

Current human evidence suggests that GLP-1 modulates reward processing primarily through cortico-striatal connectivity rather than localized changes within the NAc, with potential translational implications for obesity and addiction. Further harmonized neuroimaging studies incorporating direct pharmacological manipulation are needed to clarify NAc-specific GLP-1 mechanisms.

**Systematic review registration:**

https://www.crd.york.ac.uk/PROSPERO/view/CRD420251250453.

## Introduction

Glucagon-like peptide-1 (GLP-1) is a gut-derived incretin hormone that exerts multifaceted effects on glucose homeostasis, energy balance, and appetite regulation (Holst, 2019). While its peripheral actions, such as enhancing glucose-dependent insulin secretion, delaying gastric emptying, and reducing food intake are well established, increasing evidence indicates that GLP-1 also acts within the central nervous system to influence feeding behaviour and reward-related processes (Alhadeff et al., 2019). GLP-1 is produced mainly by neurons in the *nucleus tractus solitarius* (NTS) and projects widely to forebrain structures implicated in both energy homeostasis and motivated behaviour, including the hypothalamus, ventral tegmental area (VTA) and nucleus accumbens (NAc) (Vrang et al., 2007; Schulz et al., 2023). GLP-1 receptors are widely expressed in the hypothalamus, VTA, and NAc, and GLP-1 neurons in the NTS make monosynaptic connections within the VTA and NAc (Liu and Borgland, 2015). Within the VTA, GLP-1R expression has been demonstrated specifically in GABAergic neurons, which modulate dopaminergic output to the NAc. GLP-1 neurons originating in the nucleus tractus solitarius (NTS) form monosynaptic projections to both the VTA and NAc, providing an anatomical substrate by which brainstem metabolic signals can influence reward circuitry (Merkel et al., 2025; Zhu et al., 2025).

The NAc, a key component of the mesolimbic reward pathway, integrates dopaminergic, glutamatergic, and peptidergic inputs to modulate motivation, reinforcement, and reward valuation (Siemsen et al., 2022). Recent studies have demonstrated that activation of GLP-1 receptors (GLP-1R) within the NAc attenuates the rewarding and motivational effects of palatable foods, alcohol, and psychostimulants, suggesting a shared neurochemical substrate linking the metabolic and hedonic regulation (Alhadeff et al., 2012; Colvin et al., 2020). The NAc GLP-1 system is considered a critical interface between energy homeostasis and reward circuitry, offering potential targets for the treatment of obesity and addictive behaviours (Jerlhag, 2023).

This systematic review aims to synthesize the current literature on GLP-1 expression and receptor signalling in the NAc, focusing exclusively on evidence derived from human studies, with particular emphasis on its physiological and behavioural roles in reward modulation and metabolic regulation. Additionally, it examines the mechanisms through which GLP-1 signalling in NAc contributes to the integration of metabolic and motivational control and discusses the potential translational implications for metabolic and neuropsychiatric disorders.

## Methods

This systematic review was conducted in accordance with the Preferred Reporting Items for a Systematic Review and Meta-analysis (PRISMA) Statement and the AMSTAR −2 critical appraisal tool (McInnes et al., 2018; Shea et al., 2017). Ethical approval from an institutional review board was not obtained due to the nature of this study. The review protocol has been registered at Prospero (reference: CRD420251250453).

### Selection criteria

Inclusion criteria consisted of all original articles conducted in humans (excluding systematic reviews and case series with fewer than 12 patients). No exclusion criteria based on the publication language or date were applied.

### Search strategy

A systematic search was performed in three databases: PubMed, Scopus, and Web of Science, in December 2025. The query is shown in Supplementary Table 1.

Additionally, the references of the included primary studies and relevant available systematic reviews were screened to identify any further articles of potential interest.

### Study selection and data extraction

After duplicate removal, two authors (MG and JRN) independently participated in study selection; any disagreements were resolved by a third author (SIS). First, studies were selected based on title and abstract, and the remaining were eligible for full-text assessment. Efforts were made to contact the authors to obtain the full texts that were not publicly available. The selected studies were carefully revised to avoid repeated populations.

Data from included studies were independently extracted by two authors (MG and JRN). Data were extracted using a purpose-built form on the year of publication, country, study centre, study design, recruitment time, sample size, participants’ age, gender distribution, body mass index (BMI), and target pathology.

### Assessment of study quality

Concerning qualitative assessment, the Cochrane Risk of Bias-2 (RoB 2) tool was used for randomized clinical and crossover trials, and the National Heart, Lung, and Blood Institute (NHLBI) Study Quality Assessment Tool for observational studies (2021) (Ma et al., 2020). This assessment was independently conducted by two authors (MG and JRN), and when disagreements arose, decisions were reached by mutual consensus following a third-party review (SIS). The quality of evidence for the included articles was evaluated using the Grading of Recommendations, Assessment, Development, and Evaluation (GRADE) approach. Articles were classified into four levels of quality (high, moderate, low, and very low) (Schunemann et al., 2023).

## Results

### Search results

**Figure 1:**
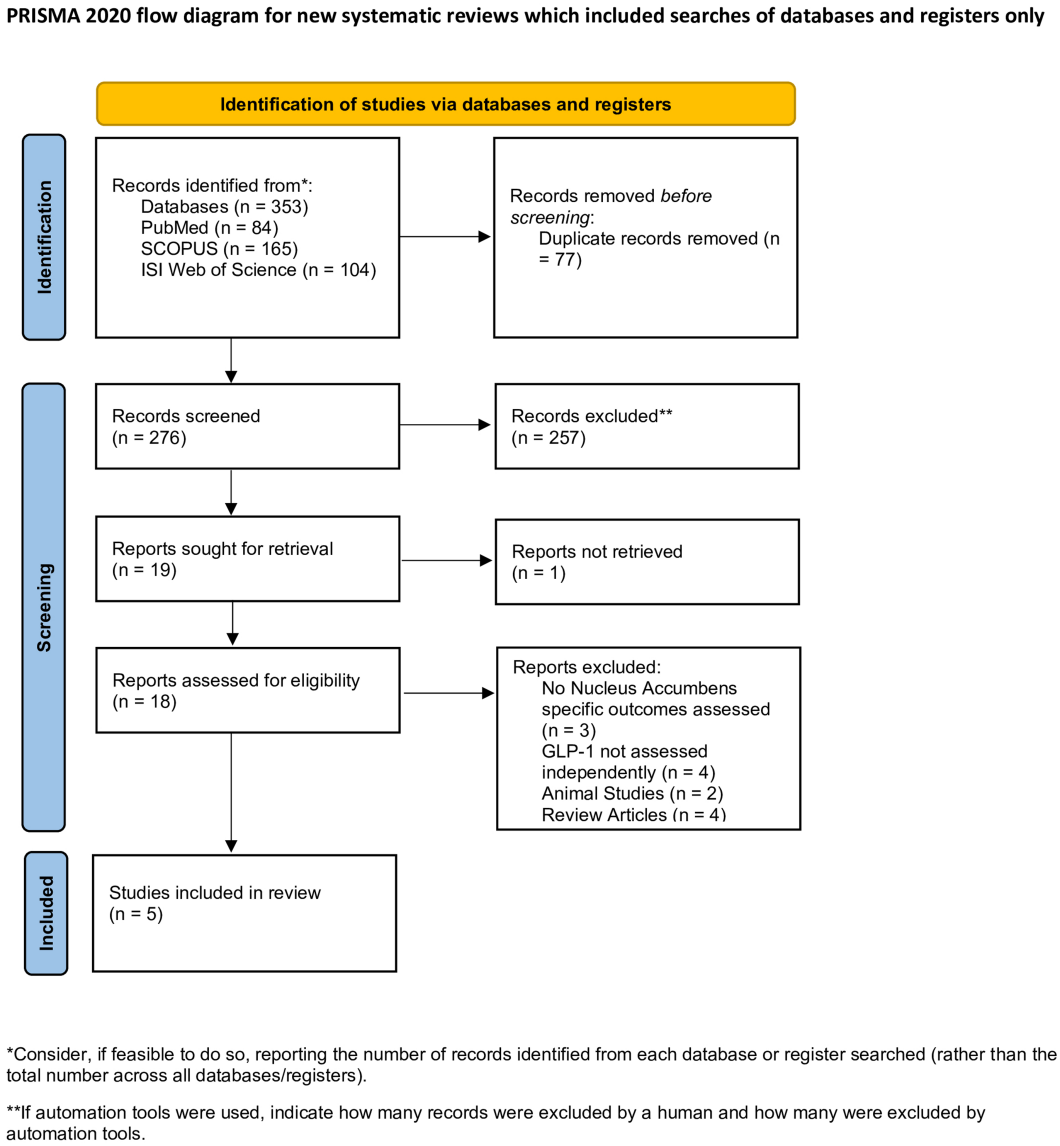
PRISMA flow diagram (McInnes et al., 2018).

After the database search and duplicate exclusion, 276 studies were screened. After selection based on title and abstract, 257 studies were excluded. Nineteen studies were sought for retrieval, of which one could not be obtained. Consequently, 18 were eligible for full-text assessment, and 13 were excluded during this process. Comprehensive reasons for exclusion upon full-text assessment were: no NAc-specific outcomes assessed (*n =* 3), GLP-1 not assessed independently (*n =* 4), animal studies (*n =* 2), and review articles (*n =* 4). Thus, a total of 5 published articles were included in this systematic review (Figure 1).

### Characteristics of included studies

As shown in Table 1, of the five studies included in this systematic review, two were cross-sectional observational studies (Dorton et al., 2017; Meng et al., 2018). One comprised two distinct components: a secondary analysis of data derived from four independent placebo-controlled human laboratory experiments and a *post-mortem* case–control study (Farokhnia et al., 2022). The remaining two studies were randomized interventional trials: one a randomized clinical trial (RCT) (Klausen et al., 2022) and one a randomized crossover trial (Meyer-Gerspach et al., 2018). The included publications were performed in six different countries within four continents: two from North America (Dorton et al., 2017; Farokhnia et al., 2022), two from Europe (Klausen et al., 2022; Meyer-Gerspach et al., 2018), one from Oceania (Farokhnia et al., 2022), and one study from Asia (Meng et al., 2018).

**Table 1:** Characteristics of included studies.

Author	Journal	Publication year	Study design	Study center	Recruitment time	Sample size	Grade
Dorton et al. (2017)	Frontiers in Psychiatry	2018	Cross-sectional observational study	University of Southern California, Los Angeles, California, USA	NA	22	**★★☆☆**
Farokhnia et al. (2022)	Addiction Biology	2022	Secondary analysis of human laboratory experiments and a post-mortem case control study	NIAAA, NIH Clinical Center, Bethesda, Maryland, USA; and NSWBTRC, University of Sydney, Australia	Not reported	69 (42 human laboratory + 27 post-mortem)	**★★☆☆**
Klausen et al. (2022)	JCI Insight	2022	Randomized, double-blinded, placebo-controlled clinical trial	Psychiatric Centre Copenhagen (Rigshospitalet) and affiliated alcohol outpatient clinics, Copenhagen, Denmark	August 7, 2017 – October 1, 2019	127	**★★★☆**
Meng et al. (2018)	Brain Imaging and Behaviour	2018	Cross-sectional case–control study	Center for Brain Imaging, Xidian University; and Tangdu Hospital, Fourth Military Medical University, Xi’an, Shaanxi, China	NA	54	**★★☆☆**
Meyer-Gerspach et al. (2018)	Diabetes, Obesity and Metabolism	2018	Randomized, cross-over trial	Research Unit, University Hospital of Basel; St. Clara Research Ltd., Basel, Switzerland; Catholic University of Leuven, Leuven, Belgium	3 months (March 2012 – June 2012)	12	**★★☆☆**

NA, Not available/not applicable; NIH, National Institutes of Health; NIAAA, National Institute on Alcohol Abuse and Alcoholism; NSWBTRC, New South Wales Tissue Resource Centre; USA, United States of America.

A total of 284 patients were assessed, ranging from 12 to 127 per study. The mean age of the participants was 41.95 years. The percentage of male participants was 65.5% (*n =* 186). Demographic and comorbidities data for the populations included in the studies were collected and are presented in Table 2.

**Table 2:** Demographic and clinical characteristics of participants.

Author	Age (mean)	Sex *n* (%)	BMI (mean)	Obesity *n* (%)	Target pathology
Dorton et al. (2017)	21.2 years	12 (54.55%) Female, 10 (45.45%) Male	22.6 kg/m^2^	NA	None (Healthy lean adults)
Farokhnia et al. (2022)	Study 1: 40.65 years Study 2: AUD 50.55 years, Controls 49.94 years	Study 1: 7 (16.67%) Female, 35 (83.33%) Male Study 2: 27 (100%) Male	Study 1: ~25–28 kg/m^2^ Study 2: AUD 24.6 28 kg/m^2^; Controls 33.0 kg/m^2^	NA	AUD
Klausen et al. (2022)	Placebo: 52.5 years; Exenatide: 52.1 years	Placebo: 26 (40.0%) Female, 39 (60.0%) Male Exenatide: 25 (40.3%) Female, 37 (59.7%) Male	Placebo 26.7 kg/m^2^ Exenatide 26.7 kg/m^2^	Total sample: 30 (23.6%)	AUD (DSM-V; ICD-10 Alcohol Dependence)
Meng et al. (2018)	OB: 28.12 years; NW: 25.50 years	OB: 15 (57.7%) Female, 11 (42.3%) Male NW: 13 (46.4%%) Female, 15 (53.6%) Male	OB: 38.40 kg/m^2^; NW: 20.68 kg/m^2^	26 (48.14% of total sample)	Obesity
Meyer-Gerspach et al. (2018)	24.8 years	12 Male (100%), 0 Female	22.9 kg/m^2^	NA	None (healthy lean males)

NA, Not available/not applicable; n, number of people; AUD, Alcohol Use Disorder; BMI, Body Mass Index; NW, Normal Weight (BMI 18.5–24.9 kg/m^2^); OB, Obese (BMI > 30 kg/m^2^).

### GLP-1 assessment

Across the included studies, GLP-1 signalling was assessed using heterogeneous methodological approaches, including peripheral hormonal measurements and post-mortem molecular quantification, as detailed in Table 3.

**Table 3:** GLP-1 assessment and intervention characteristics.

Author	GLP-1RA type	Hormonal assessment	Hormonal manipulation	Mode of administration (IV/SC/ oral/intragastric)	Acute vs. chronic exposure	Hormone dose	Duration of exposure	Control group
Dorton et al. (2017)	NA	Endogenous GLP1 was assessed in the plasma at baseline (fasting) and ~75 min after an acute oral glucose load (75 g).	NA	Oral glucose challenge	Acute	NA	Single session acute metabolic challenge; postprandial assessment at ~75 min	Within-subject water condition (flavouring water)
Farokhnia et al. (2022) –*Study 1*	NA	Endogenous active GLP-1was measured in plasma at baseline and after acute alcohol administration.	NA	Study 1: Alcohol administration (oral and IV)	Study 1: Acute (hours)	NA	Study 1: Session-based (~120 min; repeated sampling)	Study 1: NA
Farokhnia et al. (2022) –*Study 2*	NA	GLP-1 receptor mRNA expression was quantified in the *nucleus accumbens* and other brain regions using qRT-PCR.	NA	NA	Chronic AUD exposure	NA	Chronic AUD (years; post-mortem)	non-AUD
Klausen et al. (2022)	Exenatide	Plasma exenatide level and anti-exenatide antibody level	NA	SC	Chronic	2 mg once weekly	26 weeks	Placebo
Meng et al. (2018)	NA	NA	NA	NA	NA	NA	NA	Normal weight participants
Meyer-Gerspach et al. (2018)	NA (GLP-1 receptor antagonist Exendin (9–39))	Assessment: endogenous gastrointestinal hormones were measured in plasma using serial blood sampling before and after intragastric glucose administration.	Manipulation: GLP-1 receptor signalling was pharmacologically blocked by IV infusion of the GLP-1 receptor antagonist exendin.	Exendin (9–39): IV; Glucose: intragastric administration	Acute	600 pmol/kg/min	Exendin (9–39) infusion: 90 min;	Within-subject crossover (placebo infusion condition)

NA, Not available/not applicable; GLP-1, Glucagon-like peptide-1; GLP1-RA, Glucagon-like peptide-1 receptor agonist; mRNA, messenger Ribonucleic Acid; qRT-PCR, Quantitative Reverse Transcription Polymerase Chain Reaction; AUD, Alcohol Use Disorder; IV, Intravenous; SC, Subcutaneous.

Peripheral endogenous GLP-1 concentrations were evaluated in three studies under distinct experimental conditions. One study measured circulating GLP-1 levels at baseline (fasting) and approximately 75 min following an acute oral glucose load (75 g) within a single-session acute metabolic challenge paradigm (Dorton et al., 2017).

Two independent studies were conducted within the same publication (Farokhnia et al., 2022). In Study 1, circulating active GLP-1 concentrations were assessed as part of a secondary analysis of data collected from four independent human laboratory experiments. The parent studies were placebo-controlled pharmacological experiments testing different medications; however, only the placebo conditions were included to avoid potential confounding effects of active treatments.

All experiments involved acute alcohol administration to nontreatment-seeking, heavy-drinking adults. Plasma active GLP-1 was measured at multiple time points before and after alcohol exposure, under both oral and intravenous administration conditions (Farokhnia et al., 2022). Notably, plasma GLP-1 concentrations were quantified retrospectively for the present study and were not available at the time of publication of the original parent trials.

In Study 2, Central *GLP-1R* mRNA expression was quantified in post-mortem human brain tissue obtained from individuals with Alcohol Use Disorder (AUD) and matched controls. Quantitative reverse transcription polymerase chain reaction (RT-qPCR) was used to assess *GLP-1R* gene expression in the NAc and other brain regions, including the VTA, hippocampus, prefrontal cortex, and amygdala (Farokhnia et al., 2022).

Additionally, endogenous gastrointestinal hormones, including GLP-1, were assessed through serial blood sampling performed before and after intragastric glucose administration in one study (Meyer-Gerspach et al., 2018). Hormonal assessment was performed during an acute experimental session with predefined sampling intervals relative to glucose infusion.

### GLP-1 manipulation

Pharmacological manipulation of GLP-1 signalling was implemented in two studies using distinct approaches, including both receptor activation and blockade, as shown in Table 3.

One study administered GLP-1 receptor agonist (GLP-1RA) exenatide subcutaneously at a dose of 2 mg once weekly over a 26-week treatment period, within a randomized, placebo-controlled design (Klausen et al., 2022). Plasma exenatide and anti-exenatide antibody levels were monitored during the intervention phase to assess treatment exposure. In contrast, another study employed acute pharmacological blockade of GLP-1 receptors via an intravenous infusion of Exendin (9–39), a GLP-1 receptor antagonist, at 600 pmol/kg/min for 90 min (Meyer-Gerspach et al., 2018). This intervention was conducted within a subject crossover design, combined with intragastric glucose administration.

### Characteristics of reward paradigms and behavioural assessment

The reward domain investigated across the included studies is summarized in Table 4. Two studies examined food-related reward processing (Dorton et al., 2017; Meyer-Gerspach et al., 2018), two focused on alcohol-related reward (Farokhnia et al., 2022; Klausen et al., 2022), and one study investigated intrinsic functional network organization in obesity without implementing a task-based reward paradigm (Meng et al., 2018).

**Table 4:** Reward and behavioural outcomes across studies.

Author	Type of addiction	Reward type	Reward phase	Behavioural assessment	Correlation between changes in the NAc and behaviour
Dorton et al. (2017)	NA	Food Reward	Anticipation (cue-reactivity)	Hunger ratings (VAS 1–10), dietary intake assessment (24 h).	No significant correlations between NAc activity and hunger ratings were reported
Farokhnia et al. (2022)	Alcohol	Alcohol	Consumption	Clinical alcohol-related measures (AUD diagnosis, AUDIT, TLFB)	Exploratory analyses did not identify significant correlations between nucleus accumbens GLP-1R expression and alcohol-related behavioural measures.
Klausen et al. (2022)	Alcohol	Alcohol	Anticipation (cue-reactivity/incentive salience)	Change in Heavy drinking days via TLFB (primary outcome), total alcohol consumption, DUDIT	No significant direct NAc–behaviour correlations reported
Meng et al. (2018)	NA	Intrisic reward network	Resting state	Self-report psychometric scales YFAS; hunger/fullness ratings prior to imaging; HAMD, HAMA and hunger ratings.	NA
Meyer-Gerspach et al. (2018)	NA	Food Reward	Satiety/Feedback	VAS for hunger, prospective food consumption, satiety, and fullness	No direct NAc–behaviour correlation analysis reported

NA, Not available/not applicable; AUD, Alcohol Use Disorder; GLP-1, Glucagon-like Peptide-1; GLP-1R, Glucagon-like Peptide-1 Receptor; NAc, Nucleus Accumbens; AUDIT, Alcohol Use Disorders Identification Test; DUDIT, Drug Use Disorders Identification Test; HAMD, Hamilton Depression Rating Scale; HAMA, Hamilton Anxiety Rating Scale; TFLB, Time-Line Follow Back; VAS, Visual Analogue Scales; YFAS, Yale Food Addiction Scale.

The reward phase assessed varied across experimental designs. Two studies employed anticipatory cue-reactivity paradigms to evaluate neural responses to food- and alcohol-related cues, respectively (Dorton et al., 2017; Klausen et al., 2022). One assessed alcohol-related behaviour within consumption and self-administration paradigms during controlled laboratory alcohol exposure (Farokhnia et al., 2022). Another study examined postprandial responses within a satiety/feedback context following intragastric glucose administration (Meyer-Gerspach et al., 2018). In contrast, one study used resting-state functional magnetic resonance imaging (fMRI) to evaluate intrinsic network connectivity without exposure to external reward stimuli (Meng et al., 2018).

Behavioural assessments varied across studies and included subjective hunger ratings and alcohol-related measures as detailed in Table 4.

No study reported a statistically significant direct association between NAc GLP-1-related measures and behavioural outcomes (Table 4). Although exenatide reduced heavy drinking days in individuals with AUD, this clinical improvement was not directly linked to significant NAc activation changes.

Similarly, in food-related paradigms, NAc measures were not significantly correlated with hunger ratings or subjective appetite measures.

### Effects of GLP-1 signalling on nucleus accumbens activity

Across the included studies, NAc-related measures were evaluated using task-based fMRI paradigms and resting-state functional connectivity analyses (Table 5).

**Table 5:** Effects of GLP-1 signalling on nucleus accumbens activity and functional connectivity.

Author	Method used	Signal change in NAc	GLP-1 effect in NAc	Direction of effect (↑ / ↓ vs control)	Functional impact	Timepoint of outcome assessment	Dopaminergic pathway evaluated (yes/no)	Other affected nuclei	Functional connectivity (Orbitofrontal cortex; Amygdala; Hypothalamus; Midbrain / VTA)
Dorton et al. (2017)	Task-based fMRI (food-cue task; ROI analysis) + oral glucose challenge (75 g) + plasma GLP-1 measurement + 24 h dietary recalls	Trend-level increase in NAc reactivity associated with added sugar intake; became significant after adjustment for covariates. The primary significant finding observed in the dorsal striatum	No significant association between postprandial endogenous GLP-1 response and NAc food-cue reactivity was reported.	Positive association (trend) between habitual added sugar intake and nucleus accumbens food-cue reactivity after glucose ingestion; association not observed after water consumption	Added sugar ↑ striatal cue reactivity, and added sugar ↓ GLP-1 response	Postprandial after oral glucose; blood draw and hunger rating at ~75 min post-drink; fMRI performed after drink during the same visit	No	Dorsal Striatum	NA
Farokhnia et al. (2022)	Study 1: Plasma active GLP-1 measurement, Acute oral or IV alcohol administration Study 2: Post-mortem qRT-PCR for GLP-1R mRNA (NAc, VTA, hippocampus, PFC, amygdala)	No significant GLP-1R mRNA expression difference in the NAc	No significant GLP-1 receptor–related differences were observed in the nucleus accumbens.	No significant effect	Peripheral: Acute alcohol exposure was associated with a significant reduction in circulating active GLP-1 levels. Central: Post-mortem analysis showed increased GLP-1 receptor mRNA expression in the hippocampus in individuals with AUD, while no significant difference was observed in the NAc.	Study 1: Multiple timepoints before and after acute alcohol exposure (baseline and post-administration). Study 2: Single post-mortem assessment.	No	Post-mortem analyses revealed region-specific alterations in central GLP-1 receptor expression, with significant upregulation in the hippocampus, a trend in the prefrontal cortex, and no significant differences in the nucleus accumbens and ventral tegmental area.	NA
Klausen et al. (2022)	Weekly subcutaneous injections (exenatide 2 mg vs. placebo) + behavioural outcomes + fMRI alcohol cue reactivity task + fMRI N-back (spatial working memory) + SPECT DAT scan (subgroup)	No significant isolated NAc BOLD signal change reported	GLP-1 receptor agonism did not produce a significant effect in the NAc.	No significant effect vs. placebo	Exenatide reduced alcohol cue–reactivity in ventral striatal regions but did not have a significant effect in the NAc ROI.	Baseline and week 26 (treatment end) for imaging subgroup; additional single follow-up visit 6 months after treatment (clinical outcomes).	Yes	Ventral striatum (including caudate), dorsal striatum, putamen, septal area	NA
Meng et al. (2018)	Resting-state fMRI, graph theory network analysis (Brainnetome Atlas 246 ROIs; Pearson correlations; sparsity thresholding 10–30%), permutation testing and network-based statistics (NBS); fasting and pre-scan ratings; peripheral hormone assays from blood samples (Bio-Plex 200 suspension array).	NA	NA	Reduced nucleus accumbens network properties in obesity, with lower nodal degree in the right NAc and lower nodal efficiency in both left and right NAc compared with normal-weight controls.	Obesity was associated with disrupted small-world organization and reduced global integration (↑ shortest path length; ↓ global efficiency), with regional reductions in nodal degree/efficiency, including the nucleus accumbens within the frontal–mesolimbic network.	Single assessment: 12-h overnight fast; hunger/fullness rated before imaging; MRI performed in the morning between 9–10 a.m.; blood samples taken in the obese group (single baseline sampling; timing relative to scan not further specified).	No	Frontal: medial OFC (mOFC), rostral ACC (rACC), subgenual ACC (sgACC), inferior frontal gyrus (IFG). Striatal: caudate (↑ nodal degree), nucleus accumbens (↓ nodal degree/efficiency). Limbic/other: insula, amygdala, hippocampus/parahippocampal gyrus, thalamus (generally ↓ nodal metrics).	Network-based statistics identified a disrupted sub-network with reduced functional connectivity in obese subjects, predominantly involving the right rostral anterior cingulate cortex and its connections with the right lateral orbitofrontal cortex and limbic regions including the amygdala and hippocampus/ parahippocampal gyrus. Functional connectivity involving the hypothalamus and midbrain/VTA was not reported.
Meyer-Gerspach et al. (2018)	Resting-state fMRI (seed-based rsFC analysis); intragastric glucose challenge; intravenous GLP-1R antagonism with exendin (9–39); serial blood sampling for gastrointestinal hormones; VAS for appetite sensations;	NA	Endogenous GLP-1 regulates postprandial NAc functional connectivity, with increased right NAc–orbitofrontal coupling observed after pharmacological GLP-1 receptor blockade	Right NAc: increased resting-state functional connectivity with the right lateral orbitofrontal cortex during GLP-1 receptor blockade (exendin(9–39) + glucose) compared with glucose alone; left NAc: no significant difference.	GLP-1R blockade altered rsFC within mesolimbic and homeostatic networks and attenuated postpandrial reductions in prospective food consumption	Blood sampling at −10, −1, +15, +60 min; intragastric glucose at t = 0; resting-state fMRI performed ~10 min after glucose administration	Yes (indirect functional assessment of mesolimbic dopaminergic circuit via VTA seed connectivity)	Hypothalamus–lateral OFC (↑ rsFC); hypothalamus–amygdala (↑ rsFC); midbrain/VTA–caudate nucleus (↓ rsFC)	Altered rsFC involving hypothalamus–OFC, hypothalamus–amygdala, right nucleus accumbens–right lateral OFC, and midbrain/VTA–caudate nucleus

NA, Not available/not applicable; GLP-1, Glucagon-like Peptide-1; GLP-1R, Glucagon-like Peptide-1 Receptor; AUD, Alcohol Use Disorder; BOLD, blood oxygen level-dependent; MRI, Magnetic Resonance Imaging; fMRI, functional magnetic resonance imaging; BOLD fMRI, blood oxygen level-dependent functional magnetic resonance imaging; qRT-PCR, Quantitative Reverse Transcription Polymerase Chain Reaction; rsFC, resting state functional connectivity; ROI, region-of-interest; SPECT, single-photon emission computed tomography; DAT, Dopamine Transporter; mRNA, messenger Ribonucleic Acid; ACC, anterior cingulate cortex; IFG, Inferior frontal gyrus; NAc, Nucleus Accumbens; OFC, orbitofrontal cortex; VTA, Ventral Tegmental Area; PFC, prefrontal cortex; IV, Intravenous; VAS, Visual Analogue Scale.

In task-based paradigms, one study investigated neural responses to food cues following acute oral glucose ingestion (Dorton et al., 2017). Although statistically significant effects were primarily identified in dorsal striatal regions, a trend-level positive association was observed between habitual added-sugar intake and NAc food-cue reactivity following glucose ingestion. No significant association was found between postprandial endogenous GLP-1 concentrations and NAc blood oxygen level-dependent (BOLD) activity (Dorton et al., 2017).

Another study examined the effects of chronic treatment with exenatide on alcohol cue-reactivity in individuals with AUD (Klausen et al., 2022). While treatment-related reductions in alcohol cue-reactivity were reported in ventral striatal regions, no statistically significant isolated change in BOLD signal was observed within the predefined NAc ROI (Klausen et al., 2022).

In resting-state analyses, one study reported increased connectivity between the right NAc and the lateral orbitofrontal cortex (lOFC) during pharmacological receptor blockade (Exendin (9–39)) compared to glucose administration alone, with no significant effects in the left NAc (Meyer-Gerspach et al., 2018). Another study found reduced nodal degree and efficiency in bilateral NAc in obese individuals using graph-theoretical analysis. However, GLP-1 signalling was not directly manipulated in that study (Meng et al., 2018).

### GLP-1 receptor expression in the nucleus accumbens

*GLP-1R* mRNA expression was examined in post-mortem brain tissue from individuals with AUD (Farokhnia et al., 2022). No significant differences in GLP-1R mRNA expression were found in the NAc between AUD and control subjects (Table 5). Region-specific alterations were observed in other areas, including increased GLP-1R expression in the hippocampus and a trend-level increase in the prefrontal cortex, but not in the NAc.

### Nucleus accumbens functional connectivity findings

One study evaluated postprandial resting-state connectivity following intragastric glucose administration, with and without pharmacological GLP-1R blockade using Exendin (9–39) (Meyer-Gerspach et al., 2018). Increased functional connectivity between the right NAc and the lOFC was observed during GLP-1R blockade compared to glucose administration alone, whereas no significant connectivity changes were reported for the left NAc (Table 5).

Intrinsic network organization was assessed using graph theoretical analysis of resting-state fMRI data in obese and normal-weight individuals (Meng et al., 2018). Obese participants exhibited reduced nodal degree in the right NAc. They also showed reduced nodal efficiency in bilateral NAc, accompanied by increased shortest path length and decreased global efficiency at the whole-brain level. GLP-1 signalling was not experimentally manipulated in this study, and no significant between-group differences in peripheral GLP-1 concentrations were reported. None of the included fMRI studies employed subregion-specific NAc masks; all ROI definitions encompassed the nucleus accumbens as a whole, without differentiation between shell and core compartments.

### Dopaminergic pathway evaluation

One study included dopamine transporter single-photon emission computed tomography imaging in a subgroup of participants to evaluate dopaminergic markers (Klausen et al., 2022). Another study examined mesolimbic circuitry using VTA-seeded resting-state functional connectivity analysis (Meyer-Gerspach et al., 2018). The remaining studies did not directly assess dopaminergic markers or dopamine-specific neuroimaging measures (Table 5). Across the included studies, no consistent evidence of GLP-1-related dopaminergic alterations within the NAc was reported.

### Direction of effect in the nucleus accumbens

As summarized in Table 5, the direction of GLP-1-related effects in the NAc varied across studies. One study reported no significant difference in GLP-1R mRNA expression in the NAc between groups (Farokhnia et al., 2022). A second study did not observe statistically significant isolated BOLD signal changes within the predefined NAc region-of-interest (ROI) following GLP-1RA treatment (Klausen et al., 2022). In the third study, a trend-level positive association between habitual added sugar intake and NAc food-cue reactivity following glucose ingestion (Dorton et al., 2017). The fourth study identified increased functional connectivity between the right NAc and the lOFC during GLP-1 receptor blockade compared with glucose administration alone (Meyer-Gerspach et al., 2018). The last study reported reduced nodal degree in the right NAc and reduced nodal efficiency in bilateral NAc in obese individuals (Meng et al., 2018). Across the included studies, no consistent directional pattern of GLP-1-related alterations in isolated NAc activation or receptor expression was observed.

### Studies quality

**Figure 2:**
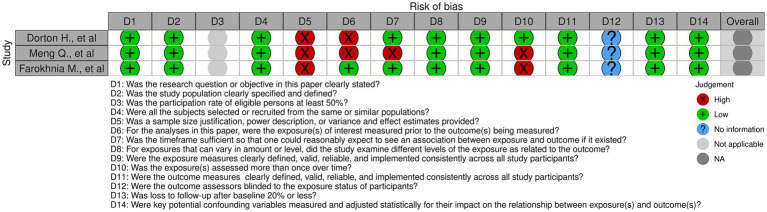
Risk of bias of observational studies.

**Figure 3:**
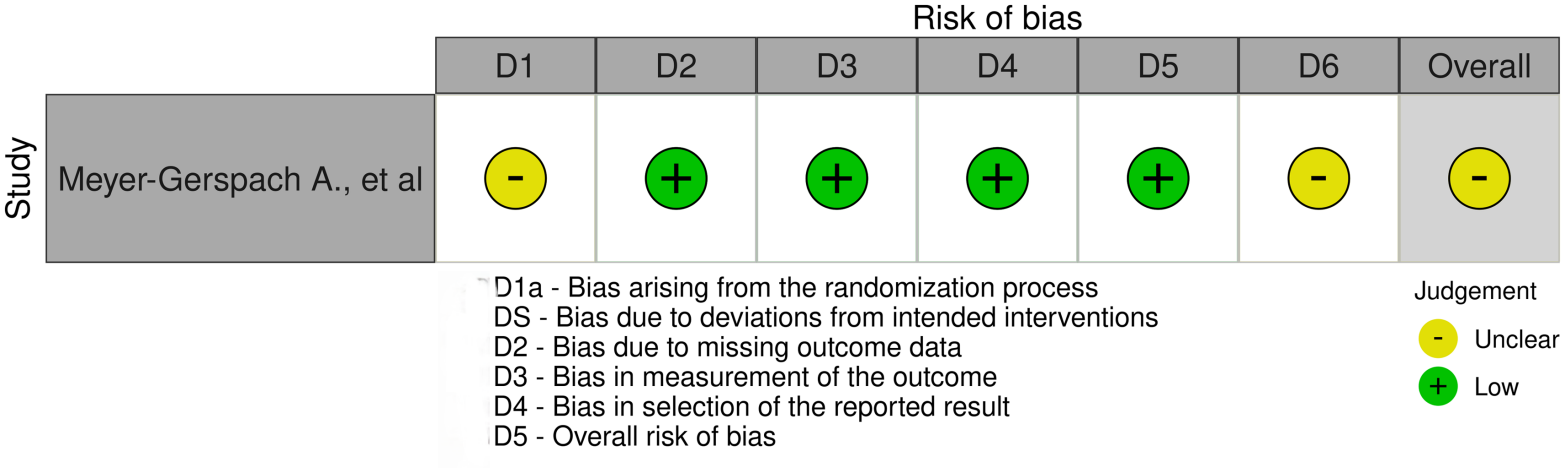
Risk of bias of the randomized crossover trial.

**Figure 4:**
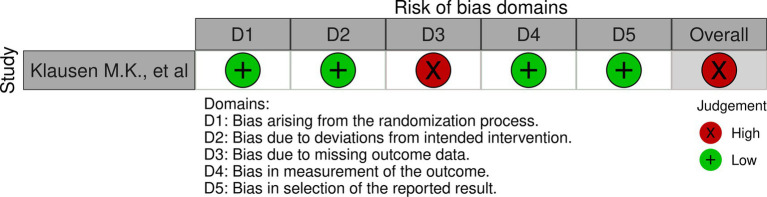
Risk of bias of the RCT.

The methodological quality of the observational studies was evaluated using the appropriate risk-of-bias domains, and the results are summarized in Figure 2, which displays the judgment for each domain across the included studies. The risk of bias of the randomized crossover trial and the RCT was assessed using the Cochrane Risk of Bias 2 tool and is illustrated in Figure 3 and Figure 4, respectively.

All observational studies had a moderate overall risk of bias. On the other hand, the RCT had a high overall risk of bias (Klausen et al., 2022), and the cross-over trial was judged as “some concerns.” The items most frequently associated with moderate risk of bias in observational studies included sample size justification, power description, variance and effect estimates, exposure assessment, measurement of key potential confounding variables, and statistical adjustment for their impact. The RCT had a high risk of bias due to missing outcome data, as a substantial proportion of participants were lost to follow-up before the primary endpoint. Because dropout in AUD trials may be associated with relapse or lack of treatment response, missingness may depend on the true outcome value and therefore bias the estimated treatment effect. According to the RoB-2 algorithm, this scenario results in a high risk of bias. The cross-over trial was judged to have some concerns because the randomization process was not adequately reported, and there was no clearly prespecified analysis plan. Specifically, the study was described as randomized, but the method used to generate the allocation sequence and details regarding allocation concealment were not reported. In addition, no prespecified statistical analysis plan was provided, raising concerns about bias arising from the randomization process and from the selection of the reported results.

## Discussion

This review synthesized current evidence regarding GLP-1 signalling and its role within the human NAc, integrating data from pharmacological interventions, functional neuroimaging, and post-mortem molecular analysis. Overall, the findings suggest that while the NAc is a theoretical hub for GLP-1-mediated integration of metabolic and reward signals, human data remains heterogeneous and scarce. While animal models consistently show robust GLP-1R activity in NAc influencing motivation, human studies assessing NAc BOLD activity report inconsistent effects, and no clear alterations in receptor expression have been identified. Notably, promising findings were more frequently observed in functional connectivity, specifically between the NAc and the lOFC, rather than in isolated NAc activation or molecular density.

The main finding of this review is that GLP-1 signalling appears to modulate the functional connectivity of NAc within the broader reward and inhibitory control circuits, rather than simply “turning on or off” the nucleus itself. Specifically, the work by Meyer-Gerspach et al. (Meyer-Gerspach et al., 2018) demonstrated that blocking GLP-1R increased connectivity between the right NAc and the lOFC. It should be noted that this finding derives from a single study conducted in healthy lean males and cannot be uncritically extrapolated to pathological populations such as those with AUD or obesity, in whom pre-existing alterations in mesolimbic circuitry may substantially modify the expression of GLP-1 signalling effects. This suggests that endogenous GLP-1 normally modulates or attenuates communication between these regions to regulate food reward. Given that the OFC plays a central role in reward valuation and decision-making, this finding may indicate that endogenous GLP-1 signalling contributes to the regulation of cortico-striatal communication involved in reward evaluation and feeding behaviour (Meyer-Gerspach et al., 2018; Rolls and Grabenhorst, 2008). The lack of significant changes in isolated NAc BOLD signal across both the RCT and observational studies suggests that GLP-1’s influence in humans might be more sophisticated than a simple reduction in reward-centre firing, acting instead as a filter on how reward signals are integrated with feedback (Meyer-Gerspach et al., 2018; ten Kulve et al., 2015). Such network-level modulation is consistent with emerging evidence suggesting that metabolic hormones influence distributed neural circuits integrating homeostatic and hedonic signals (Schulz et al., 2023; Clarke et al., 2024).

A secondary finding of interest is the apparent dissociation between NAc GLP-1R molecular expression and clinical pathology. In the post-mortem analysis by Farokhnia et al. (Farokhnia et al., 2022), individuals with AUD showed no significant differences in *GLP-1R* mRNA expression within the NAc compared to controls, despite significant alterations in other regions like the hippocampus. This suggests that the therapeutic efficacy of GLP-1RAs in treating addiction or obesity, seen in the clinical improvement in heavy drinking days reported by Klausen et al. (2022), may not depend on reversing a pre-existing molecular deficit in the NAc. Rather, these medications likely exert their effects by pharmacologically amplifying existing pathways, namely via VTA, or by acting on distal nodes of the reward circuitry that then project to the NAc (Alhadeff et al., 2012; Skibicka, 2013).

Finally, the review highlighted a complex relationship between habitual metabolic intake and NAc reactivity. The trend-level positive association between habitual sugar intake and NAc food-cue reactivity following a glucose oral load suggests that individual dietary patterns may “prime” the NAc, potentially overriding the natural inhibitory effects of postprandial GLP-1. This finding is supported by Meng et al. (2018), who reported that obese individuals exhibit reduced nodal efficiency in the NAc. Taken together, these results suggest that in states of chronic overnutrition or metabolic dysfunction, NAc may become less efficient at processing homeostatic signals, contributing to the reward deficiency or hyperreactivity to food-related cues seen in obesity and addictive behaviours (Meng et al., 2018; Green et al., 2011). However, the lack of correlation between GLP-1 levels and behavioural hunger ratings across the studies underscores the difficulty of isolating the NAc’s specific contribution to the complex, multi-organ process of human appetite regulation (Lim et al., 2023; Maurer et al., 2019).

Several limitations must be considered when interpreting the findings of this review. First, the number of human studies investigating GLP-1 signalling in the NAc remains limited, with only five articles being eligible for this systematic review, and the majority of which had small sample sizes, with a total of only 284 participants and groups as small as 12. This restricted evidence base, together with relatively small sample sizes, limits statistical power and reduces the ability to draw definitive conclusions about the role of GLP-1 within this specific brain region. In addition, substantial methodological heterogeneity was observed across the included studies. Different approaches were used to assess GLP-1 signalling, including peripheral hormone measurements, pharmacological interventions with GLP-1 receptor agonists or antagonists, post-mortem molecular analyses, and functional neuroimaging paradigms. Furthermore, the behavioural domains examined varied considerably, encompassing food reward, alcohol use, and obesity-related neural alterations. This variability limits the comparability of results and precludes a quantitative meta-analysis. Another limitation relates to the clinical heterogeneity of the included populations. AUD may occur as a primary condition or as a secondary condition to other psychiatric disorders, each associated with distinct alterations in cortico-limbic and reward circuitry (Tessner and Hill, 2010; Gilpin and Weiner, 2017; Hinostroza and Mahr, 2025). In particular, individuals with AUD and obesity present with well-documented alterations in NAc nodal efficiency and cortico-striatal communication that predate any GLP-1 intervention. These baseline differences may attenuate, amplify, or qualitatively alter GLP-1-related effects within the reward circuitry, limiting direct comparability across the included studies (Tessner and Hill, 2010; Gilpin and Weiner, 2017; Hinostroza and Mahr, 2025). Similarly, obesity includes both metabolic and eating disorder-related phenotypes with divergent neural patterns (Steward et al., 2018; Li et al., 2023). The pooling of such heterogenous subgroups may obscure GLP-1-specific effects within the NAc and broader reward networks, limiting the interpretation and generalization of the findings. The male predominance is particularly consequential given known sex differences in GLP-1 signalling, mesolimbic dopamine function, and reward processing, and substantially limits the generalisability of the present conclusions to female populations and to conditions with marked female predominance such as anorexia nervosa and binge eating disorder. Another important limitation concerns the indirect nature of many of the measurements used to infer central GLP-1 activity. In several studies, circulating GLP-1 concentrations were measured in peripheral blood, which may not accurately reflect GLP-1 signalling within central nervous system structures such as the NAc. Moreover, most neuroimaging studies relied on BOLD signal changes or functional connectivity analyses, which provide indirect measures of neuronal activity and may not capture subtle neurochemical effects of GLP-1 signalling. These limitations are further compounded by the spatial resolution constraints of current neuroimaging techniques, which often do not allow reliable distinction between the shell and core subregions of the NAc. Finally, the absence of consistent correlations between NAc neural activity and overt behavioural outcomes, together with the inherent limitations of post-mortem data, suggests that the current evidence remains fragmented. These results highlight the need for larger cohorts and imagiological studies with a primary aim to directly assess central GLP-1 signalling in humans to validate the current findings in the literature and clarify these complex neurobiological interactions.

## Conclusion

This systematic review synthesizes the current human evidence on GLP-1 signalling in the NAc and its potential role in integrating metabolic and reward-related processes. Current evidence tentatively suggests that GLP-1 may modulate reward processing via cortico-striatal connectivity rather than localised NAc changes; however, this model remains hypothesis-generating and requires confirmation in population-stratified studies across healthy and pathological reward states. These findings support a model in which GLP-1 acts not as a simple suppressor of reward processing, but as one of the neuromodulatory signals that shapes the integration of metabolic information with higher-order reward evaluation and behavioural control.

Despite the translational relevance of these findings for conditions such as obesity and addiction, the current evidence base remains constrained by small sample sizes, methodological heterogeneity, and reliance on indirect proxies of central GLP-1 activity. Moreover, the limited correspondence between neural markers and behavioural outcomes highlights the complexity of linking molecular signalling to human reward-related behaviour. Future research should prioritise larger, well-characterised cohorts and harmonised neuroimaging approaches, including high-resolution imaging capable of resolving NAc shell and core subregions, alongside pharmacological and behavioural paradigms that directly interrogate central GLP-1 signalling.

## Data Availability

The original contributions presented in the study are included in the article/supplementary material, further inquiries can be directed to the corresponding author.

## References

[ref1] Alhadeff A. L. Goldstein N. Park O. Klima M. L. Vargas A. Betley J. N. (2019). Natural and drug rewards engage distinct pathways that converge on coordinated hypothalamic and reward circuits. Neuron 103, 891–908.e6. doi: 10.1016/j.neuron.2019.05.050, 31277924 PMC6728176

[ref2] Alhadeff A. L. Rupprecht L. E. Hayes M. R. (2012). GLP-1 neurons in the nucleus of the solitary tract project directly to the ventral tegmental area and nucleus accumbens to control for food intake. Endocrinology 153, 647–658. doi: 10.1210/en.2011-1443, 22128031 PMC3275387

[ref3] Clarke G. S. Page A. J. Eldeghaidy S. (2024). The gut-brain axis in appetite, satiety, food intake, and eating behavior: insights from animal models and human studies. Pharmacol. Res. Perspect. 12:e70027. doi: 10.1002/prp2.70027, 39417406 PMC11483575

[ref4] Colvin K. J. Killen H. S. Kanter M. J. Halperin M. C. Engel L. Currie P. J. (2020). Brain site-specific inhibitory effects of the GLP-1 analogue Exendin-4 on alcohol intake and operant responding for palatable food. Int. J. Mol. Sci. 21:9710. doi: 10.3390/ijms21249710, 33352692 PMC7766977

[ref5] Dorton H. M. Luo S. Monterosso J. R. Page K. A. (2017). Influences of dietary added sugar consumption on striatal food-cue reactivity and postprandial GLP-1 response. Front. Psych. 8:297. doi: 10.3389/fpsyt.2017.00297, 29403396 PMC5777392

[ref6] Farokhnia M. Browning B. D. Crozier M. E. Sun H. Akhlaghi F. Leggio L. (2022). The glucagon-like peptide-1 system is modulated by acute and chronic alcohol exposure: findings from human laboratory experiments and a post-mortem brain study. Addict. Biol. 27:e13211. doi: 10.1111/adb.13211, 36001436 PMC12258501

[ref7] Gilpin N. W. Weiner J. L. (2017). Neurobiology of comorbid post-traumatic stress disorder and alcohol-use disorder. Genes Brain Behav. 16, 15–43. doi: 10.1111/gbb.12349, 27749004 PMC5477640

[ref8] Green E. Jacobson A. Haase L. Murphy C. (2011). Reduced nucleus accumbens and caudate nucleus activation to a pleasant taste is associated with obesity in older adults. Brain Res. 1386, 109–117. doi: 10.1016/j.brainres.2011.02.071, 21362414 PMC3086067

[ref9] Hinostroza F. Mahr M. M. (2025). The implementation of the biopsychosocial model: individuals with alcohol use disorder and post-traumatic stress disorder. Brain Behav. 15:e70230. doi: 10.1002/brb3.70230, 39740784 PMC11688116

[ref10] Holst J. J. (2019). The incretin system in healthy humans: the role of GIP and GLP-1. Metabolism 96, 46–55. doi: 10.1016/j.metabol.2019.04.014, 31029770

[ref11] Jerlhag E. (2023). The therapeutic potential of glucagon-like peptide-1 for persons with addictions based on findings from preclinical and clinical studies. Front. Pharmacol. 14:1063033. doi: 10.3389/fphar.2023.1063033, 37063267 PMC10097922

[ref12] Klausen M. K. Jensen M. E. Moller M. Le Dous N. Jensen A. O. Zeeman V. A. . (2022). Exenatide once weekly for alcohol use disorder investigated in a randomized, placebo-controlled clinical trial. JCI Insight 7, 1–15. 10.1172/jci.insight.159863 PMC9675448 36066977

[ref13] Li G. Hu Y. Zhang W. Wang J. Ji W. Manza P. . (2023). Brain functional and structural magnetic resonance imaging of obesity and weight loss interventions. Mol. Psychiatry 28, 1466–1479. doi: 10.1038/s41380-023-02025-y, 36918706 PMC10208984

[ref14] Lim J. J. Liu Y. Lu L. W. Sequeira I. R. Poppitt S. D. (2023). No evidence that circulating GLP-1 or PYY are associated with increased satiety during low energy diet-induced weight loss: modelling biomarkers of appetite. Nutrients 15:399. doi: 10.3390/nu15102399, 37242282 PMC10221374

[ref15] Liu S. Borgland S. L. (2015). Regulation of the mesolimbic dopamine circuit by feeding peptides. Neuroscience 289, 19–42. doi: 10.1016/j.neuroscience.2014.12.046, 25583635

[ref16] Ma L. L. Wang Y. Y. Yang Z. H. Huang D. Weng H. Zeng X. T. (2020). Methodological quality (risk of bias) assessment tools for primary and secondary medical studies: what are they and which is better? Mil. Med. Res. 7:7. doi: 10.1186/s40779-020-00238-8, 32111253 PMC7049186

[ref17] Maurer L. Mai K. Krude H. Haynes J. D. Weygandt M. Spranger J. (2019). Interaction of circulating GLP-1 and the response of the dorsolateral prefrontal cortex to food-cues predicts body weight development. Mol Metab. 29, 136–144. doi: 10.1016/j.molmet.2019.08.014, 31668385 PMC6812034

[ref18] McInnes M. D. F. Moher D. Thombs B. D. McGrath T. A. Bossuyt P. M. and the PRISMA-DTA Group . (2018). Preferred reporting items for a systematic review and meta-analysis of diagnostic test accuracy studies: the PRISMA-DTA statement. JAMA 319, 388–396. doi: 10.1001/jama.2017.19163, 29362800

[ref19] Meng Q. Han Y. Ji G. Li G. Hu Y. Liu L. . (2018). Disrupted topological organization of the frontal-mesolimbic network in obese patients. Brain Imaging Behav. 12, 1544–1555. doi: 10.1007/s11682-017-9802-z, 29318488

[ref20] Merkel R. Hernandez N. S. Weir V. Zhang Y. Caffrey A. Rich M. T. . (2025). An endogenous GLP-1 circuit engages VTA GABA neurons to regulate mesolimbic dopamine neurons and attenuate cocaine seeking. Sci. Adv. 11:5051. doi: 10.1126/sciadv.adr5051, 40009667 PMC11864183

[ref21] Meyer-Gerspach A. C. Ly H. G. Borgwardt S. Dupont P. Beglinger C. Van Oudenhove L. . (2018). Endogenous GLP-1 alters postprandial functional connectivity between homeostatic and reward-related brain regions involved in regulation of appetite in healthy lean males: a pilotstudy. Diabetes Obes. Metab. 20, 2330–2338. doi: 10.1111/dom.13369, 29790260

[ref22] Rolls E. T. Grabenhorst F. (2008). The orbitofrontal cortex and beyond: from affect to decision-making. Prog. Neurobiol. 86, 216–244. doi: 10.1016/j.pneurobio.2008.09.001, 18824074

[ref23] Schulz C. Vezzani C. Kroemer N. B. (2023). How gut hormones shape reward: a systematic review of the role of ghrelin and GLP-1 in human fMRI. Physiol. Behav. 263:114111. doi: 10.1016/j.physbeh.2023.114111, 36740132

[ref24] Schunemann H. J. Brennan S. Akl E. A. Hultcrantz M. Alonso-Coello P. Xia J. . (2023). The development methods of official GRADE articles and requirements for claiming the use of GRADE - a statement by the GRADE guidance group. J. Clin. Epidemiol. 159, 79–84. doi: 10.1016/j.jclinepi.2023.05.010, 37211327

[ref25] Shea B. J. Reeves B. C. Wells G. Thuku M. Hamel C. Moran J. . (2017). AMSTAR 2: a critical appraisal tool for systematic reviews that include randomised or non-randomised studies of healthcare interventions, or both. BMJ 358:j4008. doi: 10.1136/bmj.j4008, 28935701 PMC5833365

[ref26] Siemsen B. M. Barry S. M. Vollmer K. M. Green L. M. Brock A. G. Westphal A. M. . (2022). A subset of nucleus Accumbens neurons receiving dense and functional Prelimbic cortical input are required for cocaine seeking. Front. Cell. Neurosci. 16:844243. doi: 10.3389/fncel.2022.844243, 35281297 PMC8907444

[ref27] Skibicka K. P. (2013). The central GLP-1: implications for food and drug reward. Front. Neurosci. 7:181. doi: 10.3389/fnins.2013.00181, 24133407 PMC3796262

[ref28] Steward T. Menchon J. M. Jimenez-Murcia S. Soriano-Mas C. Fernandez-Aranda F. (2018). Neural network alterations across eating disorders: a narrative review of fMRI studies. Curr. Neuropharmacol. 16, 1150–1163. doi: 10.2174/1570159X15666171017111532, 29046154 PMC6187750

[ref29] ten Kulve J. S. Veltman D. J. van Bloemendaal L. Barkhof F. Deacon C. F. Holst J. J. . (2015). Endogenous GLP-1 mediates postprandial reductions in activation in central reward and satiety areas in patients with type 2 diabetes. Diabetologia 58, 2688–2698. doi: 10.1007/s00125-015-3754-x, 26385462 PMC4630252

[ref30] Tessner K. D. Hill S. Y. (2010). Neural circuitry associated with risk for alcohol use disorders. Neuropsychol. Rev. 20, 1–20. doi: 10.1007/s11065-009-9111-4, 19685291 PMC3580188

[ref31] Vrang N. Hansen M. Larsen P. J. Tang-Christensen M. (2007). Characterization of brainstem preproglucagon projections to the paraventricular and dorsomedial hypothalamic nuclei. Brain Res. 1149, 118–126. doi: 10.1016/j.brainres.2007.02.043, 17433266

[ref32] Zhu Z. Gong R. Rodriguez V. Quach K. T. Chen X. Sternson S. M. (2025). Hedonic eating is controlled by dopamine neurons that oppose GLP-1R satiety. Science 387:eadt0773. doi: 10.1126/science.adt0773, 40146831 PMC12009138

