# Supplementary material for: Nucleus accumbens GLP-1 signaling and its role in reward and metabolic regulation: a systematic review
Source: Front Neuroanat. 2026 Jul 16;20:1880560. doi: 10.3389/fnana.2026.1880560 (PMC13422549; doi:10.3389/fnana.2026.1880560)
Supplement: Supplementary file 1 [file Table_1.docx]

**Supplementary material**

**Table S1. Search strategy query**

| PubMed | (“Glucagon-Like Peptide-1 Receptor” OR “GLP-1” OR “Receptors, Glucagon” OR “Glucagon‐like Peptide 1” OR “Glucagon‐Like Peptide 1 Receptor Antagonist” OR “Glucagon‐Like Peptide 1 Receptor Agonist” or “Glucagon‐Like Peptide 1 Inhibitor” OR “Glucagon‐Like Peptide 1 Agonist” OR “GLP‐1 Receptor Antagonist” OR “GLP‐1 Receptor Agonist” OR “GLP‐1 Antagonist” OR "Long Acting GLP-1 Agonist" OR "Long Acting GLP-1 Receptor Agonist" OR "Long Acting Glucagon-Like Peptide-1 Receptor Agonist" OR "Dulaglutide" OR "Liraglutide" OR "Exenatide" OR "Albiglutide" OR "Semaglutide" OR "Lixisenatide") AND (“Behavior, Addictive” OR “Nucleus Accumbens” OR “Nucleus Accumbens” OR “Ventral Striatum”) |
| --- | --- |
| Scopus |  |
| Web of Science |  |
